# Well-Being at Home During Forced Quarantine Amid the COVID-19 Pandemic

**DOI:** 10.3389/fpsyt.2022.846122

**Published:** 2022-03-08

**Authors:** Elzbieta Krajewska-Kułak, Agnieszka Kułak-Bejda, Wojciech Kułak, Grzegorz Bejda, Cecylia Łukaszuk, Napoleon Waszkiewicz, Mateusz Cybulski, Andrzej Guzowski, Joanna Fiłon, Paulina Aniśko, Magda Popławska

**Affiliations:** ^1^Department of Integrated Medical Care, Medical University of Białystok, Białystok, Poland; ^2^Department of Psychiatry, Medical University of Białystok, Białystok, Poland; ^3^Department of Pediatric Rehabilitation and Center of Early Support for Handicapped Children “Give a Chance”, Medical University of Białystok, Białystok, Poland; ^4^The School of Medical Science in Białystok, Białystok, Poland; ^5^Doctoral School, Medical University of Białystok, Białystok, Poland; ^6^Students' Scientific Society, Department of Integrated Medical Care, Medical University of Białystok, Białystok, Poland

**Keywords:** pandemic, COVID-19, quarantine, anxiety, stress, depression

## Abstract

**Introduction:**

People recently or currently in forced quarantine or isolation at home have shown high levels of depression and symptoms of generalized anxiety.

**Aim of the Study:**

To assess the impact of the COVID-19 pandemic on certain aspects of people's day-to-day functioning.

**Materials and Methods:**

The study involved using an online diagnostic survey including a proprietary questionnaire, the DASS 21, and the Liebowitz Social Anxiety Scale.

**Results:**

Information about the pandemic in Poland and around the world was systematically obtained by 48.8 and 27.4% of respondents, respectively (*N* = 1,312). Whereas, 75.6% of respondents declared having knowledge about the number of infected people in Poland, only 28.7% declared having such knowledge about infections worldwide. Most often, respondents had obtained information online (65.9%). According to 45.7% of respondents, infection with COVID-19 is a major threat, and not enough has been done to reduce its spread in Poland (66.7%) or worldwide (56.1%). Respondents considered social distancing (68.3%), quarantining people arriving from abroad (63.4%), and wearing protective masks and/or gloves (60.4%) to be the most effective actions for combatting the pandemic. Most often, in compulsory quarantines, respondents surfed the Internet (48.8%) and experienced a lack of energy or fatigue (40.2%) and anxiety (54.9%). The severity of anxiety (mean = 4.6 points), stress (7.5 points), and depression (7.3 points) were within normal ranges, and the respondents could generally be included in the group showing mildly severe social phobia (57.9 points).

**Conclusions:**

Most respondents considered infection with COVID-19 to be a major threat and feared another quarantine. During quarantine, respondents most often experienced fatigue, a lack of energy, nervousness, anxiety, anger, and sadness. Despite demonstrating anxiety, stress, and depression with severity in the normal range, respondents showed no statistically significant correlation between severity and age, gender, place of residence, or level of education. Although they also showed mildly severe social phobia, only gender, not age, place of residence, or level of education, showed a statistically significant correlation with its severity.

## Introduction

Contemporary recommendations for forced isolation during epidemics follow the centuries-old tradition of protecting people from serious infectious diseases (1). Today, however, awareness of the dire consequences of isolating large numbers of people in quarantine means that such measures are taken only in the most serious of situations. Isolation due to epidemics indeed presents several challenges, including a diminished sense of control that can promote a sense of fear, largely because quarantine and social isolation restrict people's mobility, social interaction, and range of daily activities.

During the current pandemic, to limit the spread of COVID-19 infection worldwide, quarantine strategies have been introduced the world over, including short- and mid-term blockades, curfews, the cancellation of planned social events, the restriction of social gatherings and sport activities, the introduction of travel bans, and airspace and border closures (1–4). However, because most societies have never experienced such restrictions, people have associated the introduction of quarantines with the restriction of freedoms and imprisonment and even treated them as a form of punishment and condemnation. After all, social isolation is a form of quarantine with a recommendation not only to stay at home but also to avoid social contact outside the home, which implies separation from family, friends, and wider social networks, as well as disengagement from social activities (5–7).

Literature on people in quarantine conducted before the COVID-19 pandemic is rather sparse and most often concerns the SARS-CoV-1, MERS, Ebola, and influenza epidemics (8). In response, research on the scale and severity of emotional distress, including symptoms of depression and anxiety, in various countries remains necessary, especially to identify groups at a clinically severe risk of those symptoms. Indeed, staying in forced quarantine or home isolation is associated with several stressors that risk emotional problems, including severe symptoms of depression and/or generalized anxiety, insomnia, burnout syndrome (BOS), and post-traumatic stress disorders (PTSD) (9–14). In addition, people recently or currently in forced quarantine or isolation at home have shown relatively high levels of depression and symptoms of generalized anxiety, as well as a significantly higher severity of suicidal ideation and/or thoughts of self-harm than people not in quarantine (9). Beyond that, Logie and Turan (15) have shown that people diagnosed with COVID-19 may also experience rejection and stigmatization, which may most severely affect individuals who face discrimination daily (e.g., people of low socioeconomic status, refugees, immigrants, and minorities).

According to Chirico et al. (16), lockdown measures effectively curbing COVID-19 related new infections and deaths and overburden on the healthcare system. However, these measures are difficult to be maintained for a long time for economic reasons. This has an important implication because COVID-19 may exacerbate social inequities. Indeed, countries, where economic inequity is prevalent may be disadvantaged in the fight against the COVID-19 pandemic because the lockdown measures are unsustainable for a longer time.

Brooks et al. (9) have confirmed that people in quarantine or isolation at home may also sense a serious threat to their health and life, as well as worry that they may infect other people. Quarantine and isolation at home may also be associated with boredom, frustration due to the lack of personal freedom, and a sense of separation from the rest of the world, including loved ones. In addition, people in quarantine depend on the help of others to meet their basic needs, even in acquiring food, and awareness of such dependence can generate strong negative emotions that may increase if appropriate support from others is not received (9). Moreover, similarly to Logie and Turan (15), Brooks et al. (9) emphasized that people in quarantine or isolation may experience stigmatization and rejection from their immediate social environments, further intensifying their negative emotions.

In the study reported here, we decided to assess how the COVID-19 pandemic has influenced certain aspects of people's day-to-day functioning.

## Materials and Methods

All respondents were Poles. Inclusion criteria: age over 18 years, staying at forced 14 days quarantine amid the COVID-19 pandemic. Exclusion criteria: age below 18 years, no staying at forced 14 days quarantine amid the COVID-19 pandemic.

The study group comprised (*N* = 1,312) people, including 88.4% women and 11.6% men. The respondents' age ranged from 19 to 79 years; the mean age was 57.3 ± 19.1 years. Eighty-four percent of the respondents lived in the city, and 16% in the countryside. Forty-seven percent of the respondents had higher education, secondary - 37.2%, bachelor's - 6.7, and 8.5% during their studies, and 0.6% of people had primary education.

The study used a diagnostic survey using an Internet platform over 26 days (from January 3, 2021, to June 28, 2021). The questionnaire was anonymous. All data obtained during the study will be generalized and used in a scientific study. Participation in the study was voluntary. Entering the survey was tantamount to agreeing to fill in the survey. Respondents had the right to resign at any time, regardless of the survey stage.

The questionnaire consisted of an in-house questionnaire, the Depression Anxiety and stress scale (DASS-21), and The Leibowitz Social Anxiety Scale—LSAS.

The Bioethics Committee approved the study of the Medical University of Bialystok-APK.002.33.2021.

Lovibond and Lovibond developed the used version of the Depression Anxiety and stress scale (DASS-21) scale in 1995 (16–18);^1^ it consisted of 21 items into three groups of 7 articles each: depression, anxiety, and stress. The tool applies to the last seven days. The respondents assessed individual items on a scale from 0 to 3 points, where 0- never, 1 - sometimes, 2 - often, and 3 - always / almost always. In case of depression - normal this 0–9 point, mild this 10–13 point, moderate this 14–20 point, severe this 21–27, extremely severe this 28+. In case of anxiety - normal this 0–7 point, mild this 8–9 point, moderate this 10–14 point, severe this 15–19, extremely severe this 20+. In case of stress - normal this 0–14 point, mild this 15–18 point, moderate this 19–25 point, severe this 26–33, extremely severe this 34+.

The Leibowitz Social Anxiety Scale (LSAS) allowed assessing the severity of social phobia symptoms and their impact on everyday functioning (19). The respondent must read the descriptions of all the situations presented in the table. Each case answers two questions: “how much anxiety or fear do I experience in this situation” and “how much am I willing to avoid such a situation.” For fear/drug questions - 0 is none, 1 - mild, 2 - moderate, 3 - strong; in the case of avoiding situations - 0 - never, 1- sometimes, 2- often, and 3- always (16). The scoring scale: 0–29 No social anxiety; 30–49 Mild social anxiety; 50–64 Moderate social anxiety; 65–79 Marked social anxiety; 80–94 Severe social anxiety; >95 Very severe social anxiety.

### Statistical Analysis

All statistical analysis was performed with Statistica PL 13.0. Results are presented as mean values ± SD. Non-parametric Wilcoxon test was applied to compare differences. Spearman's analysis was used to measure the dependence age, sex, place residence, education, and the severity of depression, stress, and anxiety symptoms in the DASS 21 scale. The critical level for all tests of significance was *p* < 0.05.

## Results

Information on the COVID-19 pandemic in Poland was systematically interested in 48.8% of respondents. In turn, 27.4% of respondents were systematically interested in information about the world's coronavirus pandemic.

Almost 76% of respondents declared knowing the number of infected people in Poland, only 28.7% declared having such knowledge about infections worldwide. Most often, respondents had obtained information online (65.9%).

According to 45.7% of respondents, infection with COVID-19 is a major threat, and not enough has been done to reduce its spread in Poland (66.7%) or worldwide (56.1%).

Fifty percent of respondents reported the probability of infection with the COVID-19.

Almost 67% of Poland respondents reported that not enough had been done to protect the country against the coronavirus epidemic. Nearly 20% of respondents expressed the opposite opinion. Respondents considered social distancing (68.3%), quarantining people arriving from abroad (63.4%), and wearing protective masks and/or gloves (60.4%) to be the most effective actions for combatting the pandemic.

Almost a half (48.8%) of respondents preferred Internet surfing (42.7%), mobilizing and trying to do everything to protect themselves from infection, watching movies (39.6%), or reading (33.5%). Table 1 presents other indications.

**Table 1:** Methods of the behavior of respondents in a situation of forced quarantine^*^.

**Behavior of respondents**	**Very often**	**Often**	**Rarely**	**No**
Asking for advice and help from other people what to do in order not to get infected	2.4%	12.2%	36.6%	48.8%
Mobilizing and trying to do everything to protect yourself from infection	36.0%	42.7%	11.0%	10.4%
Reaching for alcohol, cigarettes, other psychoactive substances so as not to think about it	1.2%	7.3%	20.7%	70.7%
Consoling myself with the thought that it could be even worse, and for now, I am healthy	11.6%	48.2%	20.1%	20.1%
Giving up, not knowing what to do, not knowing what would happen - so I did nothing	4.9%	7.9%	23.2%	64%
Taking sedatives so as not to think about it	0.6%	3.0%	12.2%	84.1%
Praying for help from God	10.4%	23.2%	22%	44.5%
Watching movies	18.3%	39.6%	25%	17.1%
Reading	21.3%	33,5%	28.7%	16.5%
Cleaning	10.4%	29.9%	42.1%	17.7%
Watching TV	13.4%	29,9%	27.4%	29.3%
Internet surfing	33.5%	48.8%	13.4%	4.3%
Learning	15.2%	31.7%	28.7%	24.4%
Writing a thesis / doctoral / other scientific thesis	9.8%	7.9%	13.4%	68,9%
Taking care of the various distractions and moods	25.6%	47.6%	17.1%	9.8%

* *Possibility of multiple answers*.

The respondents declared that they most often spent between 8 and 12 h in front of the TV. The respondents often felt fatigue (40.2%), nervousness (39.6%), depression (37.2%), irritability (37.2%), or difficulty sleeping (32.9%). Details are presented in Table 2.

**Table 2:** Complaints occurring in respondents during their stay in forced quarantine.

**Complaints**	**Very often**	**Often**	**Rarely**	**No**
Headaches	11.6%	20.7%	33.5%	34.1%
Stomach pain	2.4%	8.5%	39.0%	50.0%
Dizziness	6.1%	9.8%	28.0%	56.1%
Difficulty falling asleep	23.2%	32.9%	17.1%	26.8%
Nervousness	19.5%	39.6%	26.8%	14.0%
Depression	26.2%	37.2%	25.0%	11.6%
Fatigue	28.0%	40.2%	21.3%	10.4%
Irritation	18.9%	37.2%	31.1%	12.8%

The quarantine evoked the following various emotions in the respondents: anxiety (54.9%), exhaustion (46.3%), anger (39.6%), and sadness (38.4%).

The severity of anxiety (mean = 4.6 points), stress (7.5 points), and depression (7.3 points) was within normal ranges, and the respondents could generally be included in the group showing mildly severe social phobia (57.9 points). The detailed results are presented in Table 3.

**Table 3:** Assessment of the respondents with the DASS 21 test.

**Answer**	**Never**	**Sometimes**	**Often**	**Always**
**Stress**				
I found it hard to wind down	17.1%	45.7%	1.8%	35.4%
I tended to over-react to situations	23.2%)	39.0%	6.1%	31.7%
I felt that I was using a lot of nervous energy	33.5%	34.1%	6.1%	26.2%
I found myself getting agitated	28.0%	48.8%	3.7%	19.5%
I found it difficult to relax	18.9%	47.6%	5.5%	28.0%
I was intolerant of anything that kept me from getting on with what I was doing	36.0%	39.6%	4.9%	19.5%
I felt that I was rather touchy	34.8%	46.3%	5.5%	13.4%
Mean 7.5 ± 2.5 points				
**Anxiety**				
I was aware of dryness of my mouth	46.3%	36.0%	3.0%	14.6%
I experienced breathing difficulty(eg, excessively rapid breathing, breathlessness in the absence of physical exertion)	56.7%	29.9%	1.8%	11.6%
I experienced trembling (e.g., in the hands)	67.7%	23.2%	3.0%	6.1%
I was worried about situations in which I might panic and make a fool of myself	45.7%	33.5%	4.9%	15.9%
I felt I was close to panic	58.5%	27.4%	3.7%	10.4%
I was aware of the action of my heart in the absence of physical exertion (eg, sense of heart rate increase, heart missing a beat)	50.6%	36.0%	3.7%	9.8%
I felt scared without any good reason	43.3%	38.4%	3.7%	14.6%
Mean 4.6.± 1.5 points				
**Depression**				
I could not seem to experience any positive feeling at all	25.0%	45.7%	1.2%	28.0%
I found difficulty to work up	14.6%	40.9%	10.4%	34.1%
I felt that I had nothing to look forward to	45.1%	29.9%	7.3%	17.7%
I felt down-hearted and blue	10.4%	43.3%	11.0%	35.4%
I was unable to become enthusiastic about anything	28.7%	51.2%	4.9%	15.2%
I felt I was not worth much as a person	43.9%	32.9%	5.5%	17.7%
I felt that life was meaningless	54.3%	25.0%	4.3%	6.5%
Mean 7.3 ± 2.4 points				

No significant relationship between age, sex, place of residence, and education and the severity of depression, stress, and anxiety symptoms in the DASS 21 test was found.

Almost half (45.1%) of the respondents had no social phobia on the LSAS scale. Mild social phobia had 16.5% of respondents, moderate phobia – 17.1%, severe social phobia – 9.9%, and very severe – 11.4% of respondents. The results are presented in Table 4.

**Table 4:** Assessment of social anxiety in respondents using the Leibowitz scale.

**Questions**	**How much you experience anxiety or**				**How willing you are to**			
	**fear in this situation?**				**avoid this situation?**			
	**None**	**Mild**	**Moderate**	**Severe**	**Never**	**Occasionally**	**Often**	**Usually**
Using a telephone in public	42.1%	35.4%	7.1%	5.5%	24.4%	36.0%	29.3%	10.4%
Participating in small groups	65.9%	25.6%	8.5%	0	54.3%	32.3%	11.0%	2.4%
Eating in public places	51.2%	29.3%	11.0%	8.5%	47.0%	28.0%	15.9%	9.1%
Drinking with others in public places.	55.5%	25.0%	11.6%	7.9%	42.7%	28.0%	14.6%	14.6%
Talking to people in authority	20.1%	35.4%	32.9%	11.6%	27.4%	37.8%	25.0%	9.8%
Acting, performing or giving a talk in front of an audience	12.8%	23.2%	32.9%	31.1%	17.7%	28.0%	30.5%	23.8%
Going to a party	42.7%	31.7%	16.5%	9.1%	39.0%	36.0%	14.0%	11.0%
Working while being observed	16.5%	39.0%	30.5%	14.0%	25.0%	37.8%	25.6%	11.6%
Writing while being observed	28.0%	40.9%	20.1%	11.0%	29.9%	39.6%	20.1%	10.4%
Calling someone you don't know very well	20.7%	38.4%	25.6%	15.2%	22.0%	40.2%	23.8%	14.0%
Talking with people you don't know very well	25.6%	36.6%	26.2%	11.6%	26.2%	44.5%	20.1%	9.1%
Meeting strangers	27.4%	39.0%	22.6%	11.0%	38.4%	34.1%	18.3%	9.1%
Urinating in a public bathroom	33.5%	26.8%	21.3%	18.3%	34.1%	22.6%	20.7%	22.6%
Entering a room when others are already seated	29.3%	36.6%	21.3%	12.8%	36.0%	32.9%	20.7%	10.4%
Being the center of attention	26.2%	28.0%	26.2%	19.5%	26.8%	29.9%	25.6%	17.7%
Speaking up at a meeting.	18.3%	25.0%	26.8%	29.9%	18.3%	31.1%	24.4%	26.2%
Taking a test	23.8%	32.9%	32.9%	10.4%	31.7%	38.4%	23.2%	6.7%
Expressing a disagreement or disapproval to people you don't know very well	22.0%	39.6%	30.5%	7.9%	23.8%	35.4%	27.4%	13.4%
Looking at people you don't know very well in the eyes	30.5%	39.0%	20.7%	9.8%	31.7%	36.6%	20.7%	11.0%
Giving a report to a group	15.2%	23.2%	36.0%	25.6%	20.7%	29.9%	28.0%	21.3%
Trying to pick up someone	19.5%	32.3%	28.7%	19.5%	28.0%	26.8%	20.1%	25.0%
Returning goods to a store	25.6%	31.7%	23.2%	19.5%	27.4%	22.6%	20.1%	29.9%
Giving a party	31.7%	36.0%	23.8%	8.5%	36.0%	37.8%	18.3%	7.9%
Resisting a high pressure salesperson	29.3%	36.6%	22.6%	11.5%	28.7%	32.9%	17.1%	21.3%

No significant relationship between the severity of social phobia and age, place of residence, and education was found. The only positive correlation between the severity of social phobia and gender (*R* = 0.16904; *p* = 0.0304) was found.

## Discussion

Due to the COVID-19 pandemic, sudden and severe restrictions influenced many people's mental health in the world. The quarantined people had to deal with stressful living conditions without prior preparation (20, 21). Each crisis or disaster pandemic carries a high risk of diminished wellbeing and individuals and societies as a whole (5, 22–24).

Hamer et Baran (22) conducted a study four times in 2020 (in March, April, at the turn of May and June, and in December) the CAWI (Computer-Assisted Web Interview) on a sample of 1,098 people aged 18 and over. They demonstrated a relatively high level of nervousness at the beginning of the pandemic in April. At the turn of May and June, a significant decrease was the lowest compared to the remaining months, then increased again to the level from April in December.

In a study from China (24), most respondents spent 20–24 h a day (84.7%) at home. In a study by Huang and Zhao (21), in a group of 603 randomly selected respondents, 264 people spent more than 3 h each day tracking information about the virus and the epidemic.

Information about the pandemic in Poland and around the world was systematically obtained by 48.8 and 27.4% of respondents, respectively (*N* = 328). The respondents most often obtained information about the pandemic from the Internet (65.9%) and television (22%).

The COVID-19 pandemic is a potent stressor affecting the functioning of many countries and aggravates social stress (9).

According to 40.9% of respondents, COVID-19 is a grave threat to Poles' lives in the present study. The probability of developing the coronavirus was most often determined by fifty percent of the respondents.

In the literature (25–34) quarantine may reveal mental health problems in people who did not before. Symptoms of post-traumatic stress and emotional exhaustion are also described.

The scientific publications show that in about 33% of people in isolation, their mental wellbeing worsened, and the severity of these symptoms was individual.

The pandemic clinical picture's most typical and common feature is an acute stress disorder. According to Heitzman (34), it is a prolonged anxiety reaction and the inability to break away from trauma's constant experience.

In a study from India, 12.5% of respondents reported sleep problems and, 37.8% had thoughts related to the possibility of COVID-19 infection. Furthermore, over 80% of respondents felt the need for mental support from the health care system (3).

The respondents reported mainly fatigue, nervousness, depression, and irritability in the current study.

Our results are similar to Pierce et al. (35) in the United Kingdom. The prevalence of clinically significant mental distress levels in the population increased from 18.9% in 2018–2019 to 27.3% in April 2020, 1 month after the UK economy closed. The increases were most significant among people aged 18–34, women living with young preschool children, and working before the epidemic.

In China, the impact of quarantine on the mental state, level of anxiety, depression, and stress during the initial stage of the COVID-19 outbreak in a group of 1,210 people was assessed by Wang et al. (36). More than half (53.8%) of respondents rated the psychological impact of the COVID-19 pandemic on wellbeing as moderate or severe; 16.5% of respondents had severe depression, and 28.8% had severe anxiety symptoms. Women and students had higher levels of stress, anxiety, and depression. Lower levels of stress, anxiety, and depression positively correlated with accurate health information about the COVID-19 epidemic.

In a large study group of 52,730 respondents from 36 provinces of China, Qiu et al. (37) evaluated the impact of stress amid COVID-19. Moderate stress was found in 29% of respondents, while 5% had severe stress intensity. Women had more severe stress than men. Furthermore, the subjects aged 18–30 and over 60 and higher education levels had greater stress intensity.

Another Chinese study of 600 general population during national quarantine (25) demonstrated that women had 3.01 times higher risk of anxiety than men. Respondents over 40 years of age had a lower risk of anxiety than people under 40. The risk of depression depended on the level of education.

Similar findings were reported (29) in a 603 randomly selected respondents study. Generalized anxiety had 34% of participants, and depressive disorders - in 18.1%-were more often observed respondents 35 years of age.

In an online survey from India, Roy et al. (3) assessed the level of anxiety and level of knowledge about the course of COVID-19 using. More than 80% of surveyed had a high level of anxiety. On the other hand, most of the respondents had a moderate level of knowledge about COVID-19 and a high level of knowledge about prevention.

In the current study, the severity of anxiety, stress, and depression was within normal ranges, and the respondents could be included in the group showing mildly severe social phobia (57.9 points). In addition, most respondents considered quarantine of people coming from abroad (63.4%), and cancellation of all mass events (59.1%) as the most effective actions in the fight against the spread of the coronavirus in Poland. Also, the respondents indicated keeping a safe distance between people in public space (68.3%), protective masks and gloves when leaving the house (60.4%), frequent washing of hands with soap (59.8%), the use of special disinfectants (57.3%) and avoiding public transport (43.3%).

In the present study, very common ways of behaving in a situation of forced quarantine were surfing the Internet (48.8%), mobilizing and trying to do everything to protect yourself from infection (42.7%), watching movies (39.6%), or reading (33.5%).

Heitzman (34) noted that people who test positive for the coronavirus, who are sick or quarantined, and their families would develop acute stress disorder symptoms (308.3, DSM-5) of the nature of distress.

In some countries, expert guidance was published at the pandemic's start. For example, the Korean Neuropsychiatric Association has published guidelines based on the assumption that quarantine induced by the COVID-19 epidemic may cause severe psychological effects in acute stress disorder, depression, post-traumatic stress disorder (PTSD), insomnia, irritability, and emotional exhaustion. The guidelines mention groups that are particularly vulnerable to the psychological consequences of quarantine. Experts include parents caring for children, young children, people quarantined after contact with COVID-19, doctors dealing with infected patients (38).

A study from Brazil (20), on 1,468 volunteers via an online survey, demonstrated that people who had to work outside live with an older adult have at least one common comorbid disease experienced more significant psychological discomfort and distress during the pandemic. Conversely, children's presence protected the subjects from depression.

It is impossible to compare the data to the norms as there are no standards for measuring quarantine response. Therefore, there is a need to understand the role of behavioral and psychosocial factors in predicting mental health in people in confinement and social isolation. Heitzman (34) notes that not everyone confronted with the pandemic will reveal post-traumatic psychiatric symptoms and will need psychological help and support from others. In the available works on the topics mentioned above, it was emphasized:

- the need for special care for vulnerable groups when planning preventive psychological interventions during the COVID-19 epidemic (37)
- the need to raise awareness of the psychological consequences of this COVID-19 pandemic and to intensify preventive measures to avoid long-term consequences (3)
- the need to support groups such as young people, the elderly, women, and migrants through the healthcare system, improving telemedicine and interventions during quarantine to prevent long-term consequences in the form of mental disorders (36)
- the need to identify the weakest people who may need the most help from health care systems, which seems particularly important as the human resources of psychologists or psychiatrists are limited and should be wisely (based on reliable parameters) used to fight the consequences of the COVID-19 pandemic (20)
- the need for the state to maintain access to assistance in the event of domestic violence, but also to prioritize the availability of childcare (36)
- that obtaining and relying on reliable information about an epidemic may reduce the intensity of the anxiety response, which is expected in the situation (38)
- when planning prophylaxis and interventions, one of at least six groups should be considered—healthcare professionals, people who have direct contact with patients, patients who refuse treatment, and people susceptible to infection (39).

It is well known that women were more likely to suffer from psychological stress than men. Females are more than twice as likely as males to be afflicted by mood disorders (40). This sex disparity indicates a potential role for gonadal hormones in the etiology of anxiety and depressive disorders. Women often experience anxiety, and depression during times of hormonal flux, such as puberty, menopause, perimenstrual and post-partum periods (41). According to Bucciarelli et al. (42), study gender represents a potential modifying factor in cardiovascular disease and depression and COVID-19 short- and long-term outcomes, particularly in cases involving long-term COVID complications. Results from emerging studies indicate that the COVID-19 pandemic affected male and female populations differently. Women seem to experience less severe short-term complications but suffer worse long-term COVID complications, including depression, reduced physical activity, and deteriorating lifestyle habits, all of which may impact cardiovascular risk. Mass-quarantine, self-quarantine, and isolation are associated with depression, anger, and chronic stress. The stressor factors suggested included longer quarantine duration, frustration, boredom, inadequate supplies, inadequate information, financial loss, and stigma (43).

Our current study has some potential limitations. First, the study group was too small to generalize the results to the entire population of people in Poland. Secondly, there was an overrepresentation of women in the studied subgroups. Hence the results should be verified in an equally numerous group of men. Nevertheless, despite these limitations, this study's results may provide a starting point for further research into the problems arising from quarantine.

## Conclusions

1. Most respondents considered infection with COVID-19 to be a significant threat and feared another quarantine.
2. During quarantine, respondents most often experienced fatigue, a lack of energy, nervousness, anxiety, anger, and sadness.
3. Respondents demonstrated anxiety, stress, and depression severity in the normal range.
4. Respondents showed mildly severe social phobia.
5. Due to their frequent occurrence of anxiety disorders and depression, it is worth educating people on recognizing them to seek professional help in time (a psychologist, psychotherapist, or psychiatrist). It is important to disseminate the most important advice and tips of mental health experts during a pandemic among the public. TV and social media channels that fuel a spiral of anxiety and stress should be limited. Information should be sought from reliable sources. We have to try as much as possible to keep the current, personal way of spending time and the rhythm of the day. Do not give up on favorite activities and interests.

## Data Availability Statement

The raw data supporting the conclusions of this article will be made available by the authors, without undue reservation.

## Ethics Statement

The studies involving human participants were reviewed and approved by the Bioethics Committee of the Medical University of Białystok-APK.002.33.2021. Written informed consent for participation was not required for this study in accordance with the national legislation and the institutional requirements.

## Author Contributions

EK-K and AK-B designed the study and wrote the protocol. EK-K, AK-B, WK, GB, CŁ, NW, MC, AG, JF, PA, and MP data collection. WK undertook the statistical analysis. EK-K, AK-B, and GB wrote the first draft of the manuscript. All authors contributed to and have approved the final manuscript.

## Conflict of Interest

The authors declare that the research was conducted in the absence of any commercial or financial relationships that could be construed as a potential conflict of interest.

## Publisher's Note

All claims expressed in this article are solely those of the authors and do not necessarily represent those of their affiliated organizations, or those of the publisher, the editors and the reviewers. Any product that may be evaluated in this article, or claim that may be made by its manufacturer, is not guaranteed or endorsed by the publisher.

## Footnotes

^1^Available online at: https://www.healthfocuspsychology.com.au/tools/dass-21/ (cited 29.12.2021).
